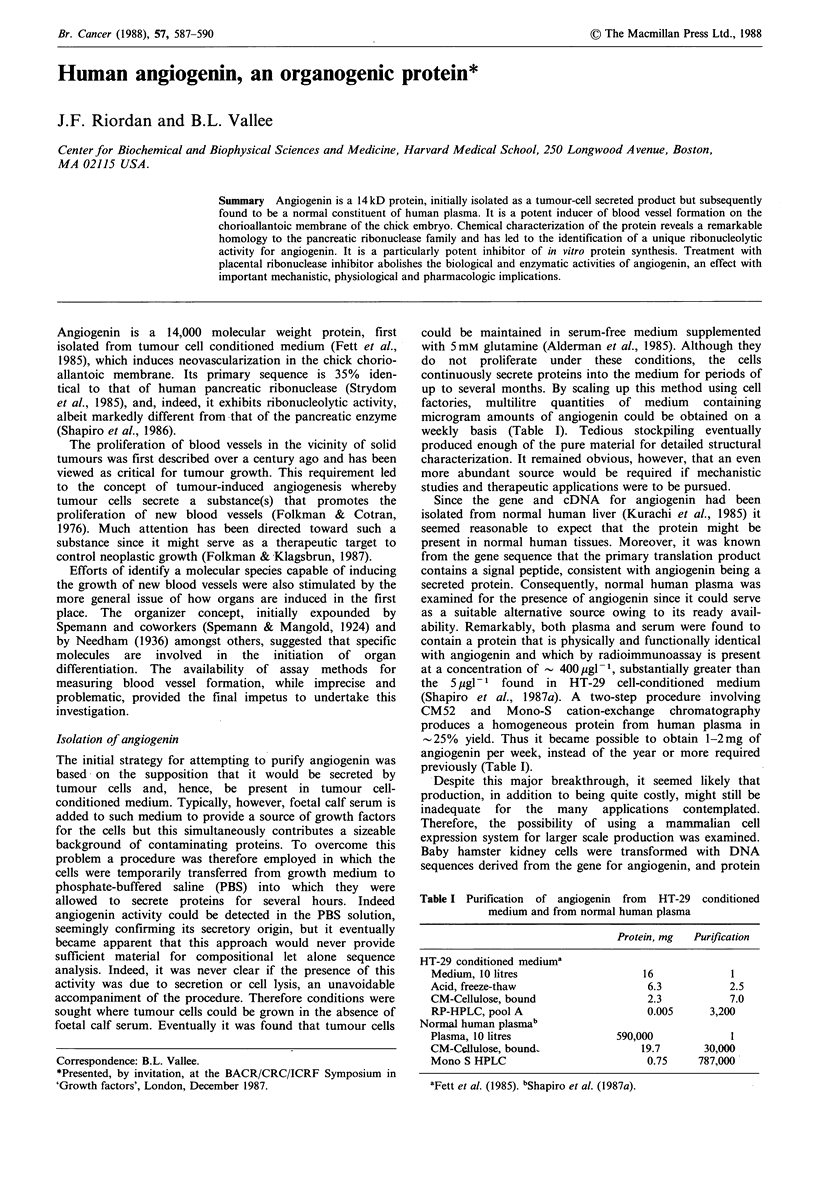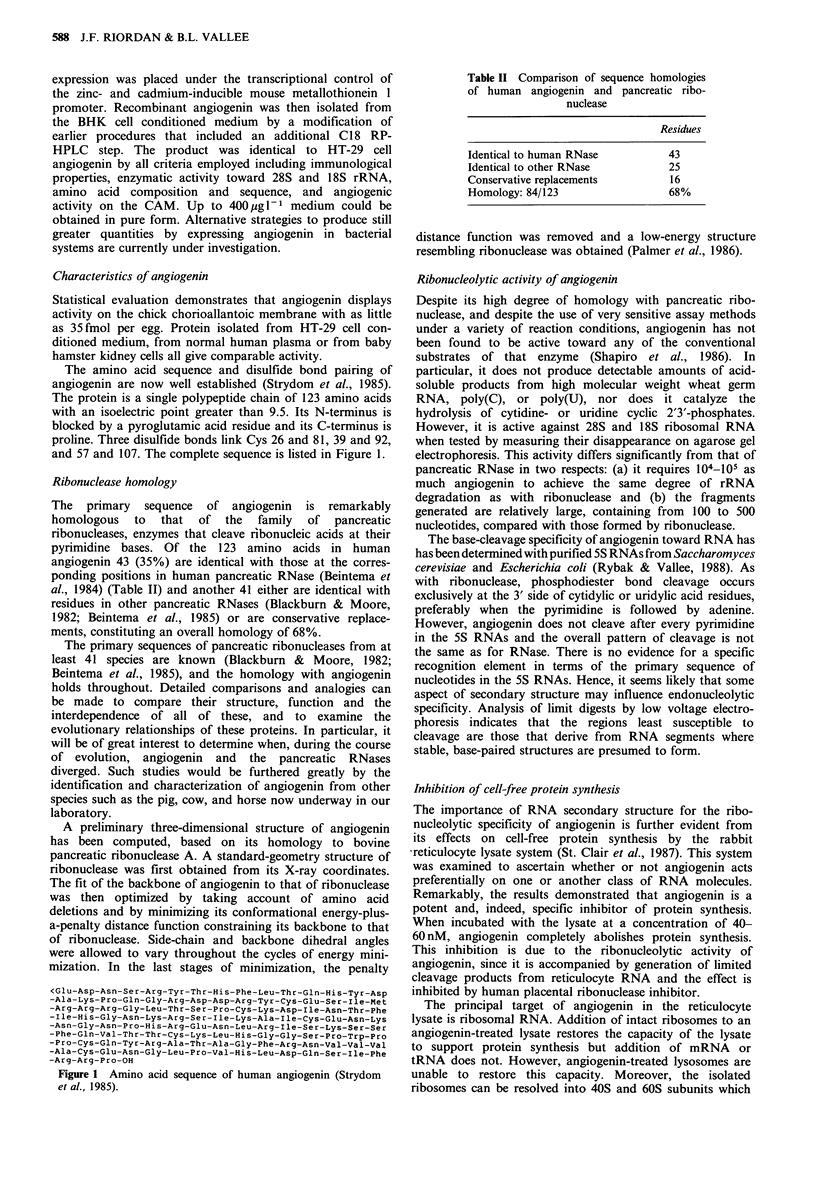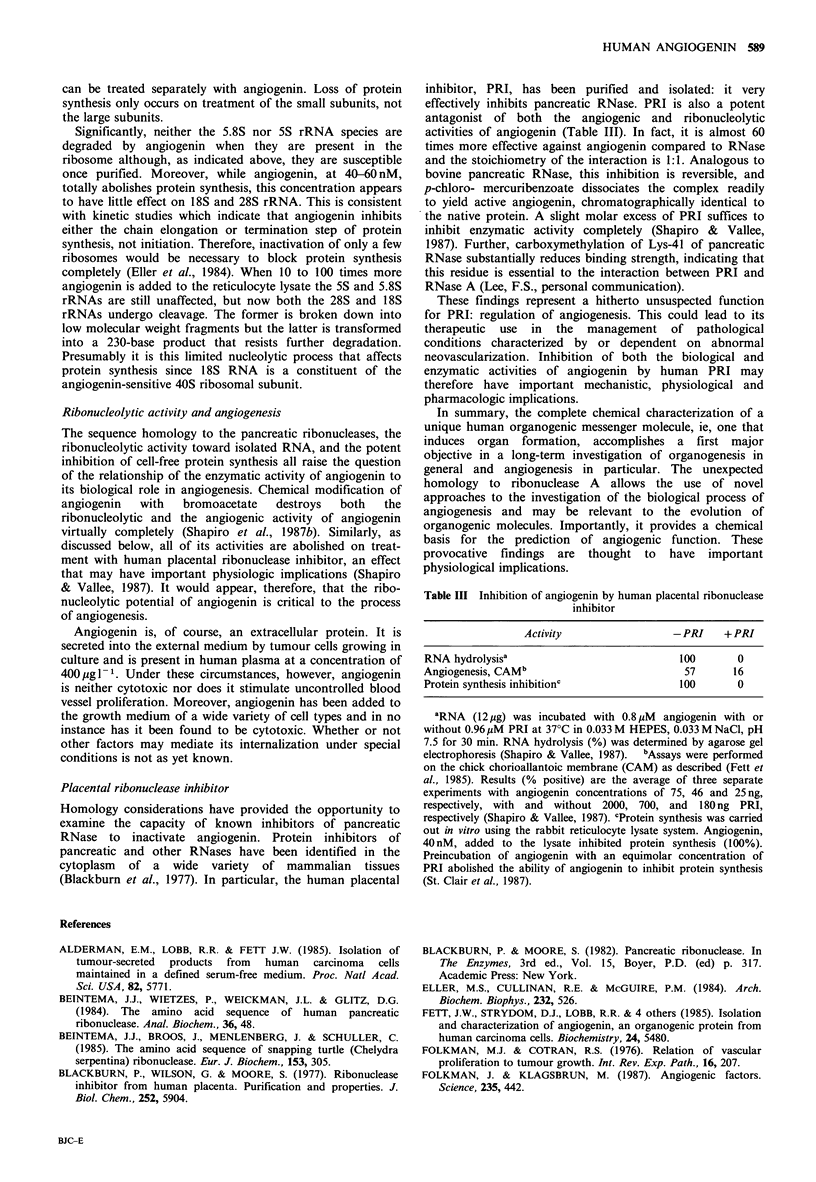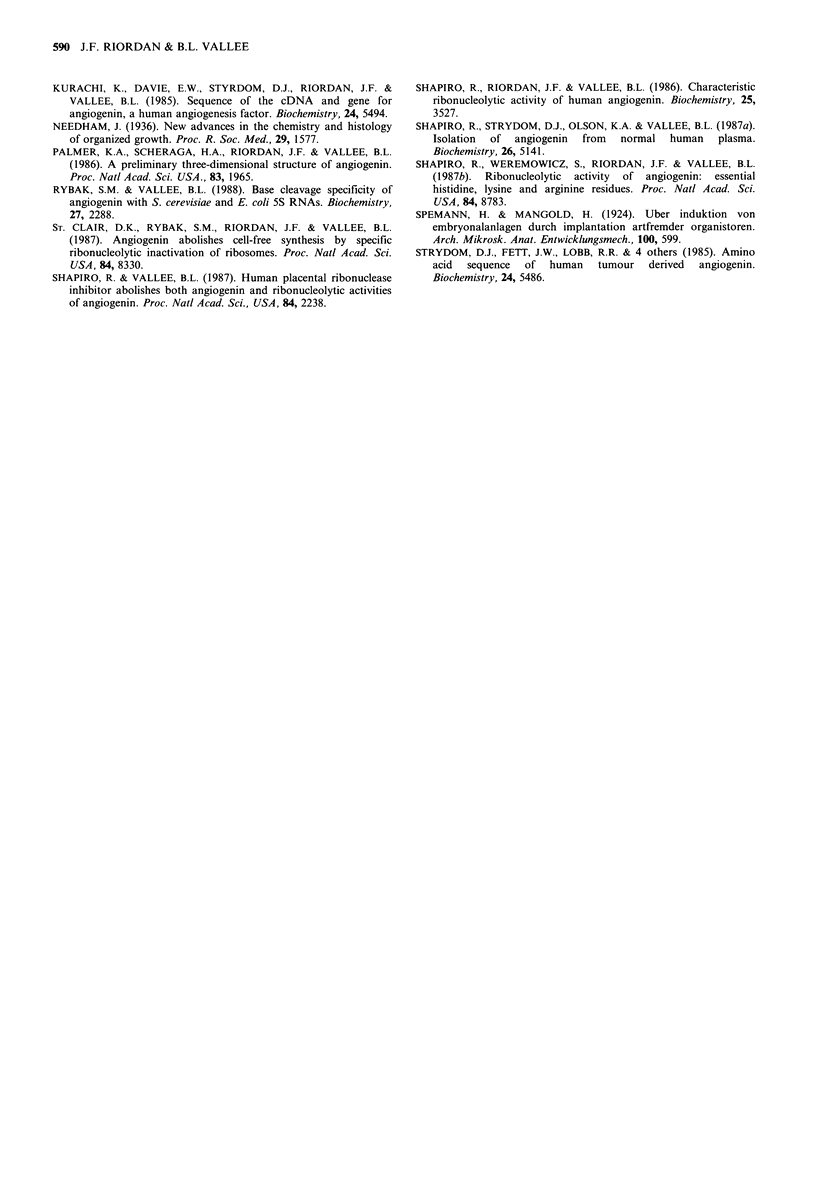# Human angiogenin, an organogenic protein.

**DOI:** 10.1038/bjc.1988.133

**Published:** 1988-06

**Authors:** J. F. Riordan, B. L. Vallee

**Affiliations:** Center for Biochemical and Biophysical Sciences and Medicine, Harvard Medical School, Boston, MA.

## Abstract

Angiogenin is a 14 kD protein, initially isolated as a tumour-cell secreted product but subsequently found to be a normal constituent of human plasma. It is a potent inducer of blood vessel formation on the chorioallantoic membrane of the chick embryo. Chemical characterization of the protein reveals a remarkable homology to the pancreatic ribonuclease family and has led to the identification of a unique ribonucleolytic activity for angiogenin. It is a particularly potent inhibitor of in vitro protein synthesis. Treatment with placental ribonuclease inhibitor abolishes the biological and enzymatic activities of angiogenin, an effect with important mechanistic, physiological and pharmacologic implications.


*Br. Cancer* (1988), **57**, 587–590 © The Macmillan Press Ltd., 1988

### Human angiogenin, an organogenic protein*

J.F. Riordan and B.L. Vallee

*Center for Biochemical and Biophysical Sciences and Medicine, Harvard Medical School, 250 Longwood Avenue, Boston, MA 02115 USA.*

**Summary** Angiogenin is a 14 kD protein, initially isolated as a tumour-cell secreted product but subsequently found to be a normal constituent of human plasma. It is a potent inducer of blood vessel formation on the chorioallantoic membrane of the chick embryo. Chemical characterization of the protein reveals a remarkable homology to the pancreatic ribonuclease family and has led to the identification of a unique ribonucleolytic activity for angiogenin. It is a particularly potent inhibitor of *in vitro* protein synthesis. Treatment with placental ribonuclease inhibitor abolishes the biological and enzymatic activities of angiogenin, an effect with important mechanistic, physiological and pharmacologic implications.

Angiogenin is a 14,000 molecular weight protein, first isolated from tumour cell conditioned medium (Fett *et al.*, 1985), which induces neovascularization in the chick chorio-allantoic membrane. Its primary sequence is 35% identical to that of human pancreatic ribonuclease (Strydom *et al.*, 1985), and, indeed, it exhibits ribonucleolytic activity, albeit markedly different from that of the pancreatic enzyme (Shapiro *et al.*, 1986).

The proliferation of blood vessels in the vicinity of solid tumours was first described over a century ago and has been viewed as critical for tumour growth. This requirement led to the concept of tumour-induced angiogenesis whereby tumour cells secrete a substance(s) that promotes the proliferation of new blood vessels (Folkman & Cotran, 1976). Much attention has been directed toward such a substance since it might serve as a therapeutic target to control neoplastic growth (Folkman & Klagsbrun, 1987).

Efforts of identify a molecular species capable of inducing the growth of new blood vessels were also stimulated by the more general issue of how organs are induced in the first place. The organizer concept, initially expounded by Spemann and coworkers (Spemann & Mangold, 1924) and by Needham (1936) amongst others, suggested that specific molecules are involved in the initiation of organ differentiation. The availability of assay methods for measuring blood vessel formation, while imprecise and problematic, provided the final impetus to undertake this investigation.

### *Isolation of angiogenin*

The initial strategy for attempting to purify angiogenin was based on the supposition that it would be secreted by tumour cells and, hence, be present in tumour cell-conditioned medium. Typically, however, foetal calf serum is added to such medium to provide a source of growth factors for the cells but this simultaneously contributes a sizeable background of contaminating proteins. To overcome this problem a procedure was therefore employed in which the cells were temporarily transferred from growth medium to phosphate-buffered saline (PBS) into which they were allowed to secrete proteins for several hours. Indeed angiogenin activity could be detected in the PBS solution, seemingly confirming its secretory origin, but it eventually became apparent that this approach would never provide sufficient material for compositional let alone sequence analysis. Indeed, it was never clear if the presence of this activity was due to secretion or cell lysis, an unavoidable accompaniment of the procedure. Therefore conditions were sought where tumour cells could be grown in the absence of foetal calf serum. Eventually it was found that tumour cells could be maintained in serum-free medium supplemented with 5 mM glutamine (Alderman *et al.*, 1985). Although they do not proliferate under these conditions, the cells continuously secrete proteins into the medium for periods of up to several months. By scaling up this method using cell factories, multilitre quantities of medium containing microgram amounts of angiogenin could be obtained on a weekly basis (Table I). Tedious stockpiling eventually produced enough of the pure material for detailed structural characterization. It remained obvious, however, that an even more abundant source would be required if mechanistic studies and therapeutic applications were to be pursued.

Since the gene and cDNA for angiogenin had been isolated from normal human liver (Kurachi *et al.*, 1985) it seemed reasonable to expect that the protein might be present in normal human tissues. Moreover, it was known from the gene sequence that the primary translation product contains a signal peptide, consistent with angiogenin being a secreted protein. Consequently, normal human plasma was examined for the presence of angiogenin since it could serve as a suitable alternative source owing to its ready availability. Remarkably, both plasma and serum were found to contain a protein that is physically and functionally identical with angiogenin and which by radioimmunoassay is present at a concentration of $\sim 400\,\mu g l^{-1}$, substantially greater than the $5\,\mu g l^{-1}$ found in HT-29 cell-conditioned medium (Shapiro *et al.*, 1987*a*). A two-step procedure involving CM52 and Mono-S cation-exchange chromatography produces a homogeneous protein from human plasma in ~25% yield. Thus it became possible to obtain 1–2 mg of angiogenin per week, instead of the year or more required previously (Table I).

Despite this major breakthrough, it seemed likely that production, in addition to being quite costly, might still be inadequate for the many applications contemplated. Therefore, the possibility of using a mammalian cell expression system for larger scale production was examined. Baby hamster kidney cells were transformed with DNA sequences derived from the gene for angiogenin, and protein

**Table I** Purification of angiogenin from HT-29 conditioned medium and from normal human plasma

| | *Protein, mg* | *Purification* |
|---|---|---|
| HT-29 conditioned medium[a] | | |
| Medium, 10 litres | 16 | 1 |
| Acid, freeze-thaw | 6.3 | 2.5 |
| CM-Cellulose, bound | 2.3 | 7.0 |
| RP-HPLC, pool A | 0.005 | 3,200 |
| Normal human plasma[b] | | |
| Plasma, 10 litres | 590,000 | 1 |
| CM-Cellulose, bound | 19.7 | 30,000 |
| Mono S HPLC | 0.75 | 787,000 |

[a]Fett *et al.* (1985). [b]Shapiro *et al.* (1987*a*).

Correspondence: B.L. Vallee.

*Presented, by invitation, at the BACR/CRC/ICRF Symposium in 'Growth factors', London, December 1987.

expression was placed under the transcriptional control of the zinc- and cadmium-inducible mouse metallothionein 1 promoter. Recombinant angiogenin was then isolated from the BHK cell conditioned medium by a modification of earlier procedures that included an additional C18 RP-HPLC step. The product was identical to HT-29 cell angiogenin by all criteria employed including immunological properties, enzymatic activity toward 28S and 18S rRNA, amino acid composition and sequence, and angiogenic activity on the CAM. Up to $400 \mu g l^{-1}$ medium could be obtained in pure form. Alternative strategies to produce still greater quantities by expressing angiogenin in bacterial systems are currently under investigation.

*Characteristics of angiogenin*

Statistical evaluation demonstrates that angiogenin displays activity on the chick chorioallantoic membrane with as little as 35 fmol per egg. Protein isolated from HT-29 cell conditioned medium, from normal human plasma or from baby hamster kidney cells all give comparable activity.

The amino acid sequence and disulfide bond pairing of angiogenin are now well established (Strydom *et al.*, 1985). The protein is a single polypeptide chain of 123 amino acids with an isoelectric point greater than 9.5. Its N-terminus is blocked by a pyroglutamic acid residue and its C-terminus is proline. Three disulfide bonds link Cys 26 and 81, 39 and 92, and 57 and 107. The complete sequence is listed in Figure 1.

*Ribonuclease homology*

The primary sequence of angiogenin is remarkably homologous to that of the family of pancreatic ribonucleases, enzymes that cleave ribonucleic acids at their pyrimidine bases. Of the 123 amino acids in human angiogenin 43 (35%) are identical with those at the corresponding positions in human pancreatic RNase (Beintema *et al.*, 1984) (Table II) and another 41 either are identical with residues in other pancreatic RNases (Blackburn & Moore, 1982; Beintema *et al.*, 1985) or are conservative replacements, constituting an overall homology of 68%.

The primary sequences of pancreatic ribonucleases from at least 41 species are known (Blackburn & Moore, 1982; Beintema *et al.*, 1985), and the homology with angiogenin holds throughout. Detailed comparisons and analogies can be made to compare their structure, function and the interdependence of all of these, and to examine the evolutionary relationships of these proteins. In particular, it will be of great interest to determine when, during the course of evolution, angiogenin and the pancreatic RNases diverged. Such studies would be furthered greatly by the identification and characterization of angiogenin from other species such as the pig, cow, and horse now underway in our laboratory.

A preliminary three-dimensional structure of angiogenin has been computed, based on its homology to bovine pancreatic ribonuclease A. A standard-geometry structure of ribonuclease was first obtained from its X-ray coordinates. The fit of the backbone of angiogenin to that of ribonuclease was then optimized by taking account of amino acid deletions and by minimizing its conformational energy-plus-a-penalty distance function constraining its backbone to that of ribonuclease. Side-chain and backbone dihedral angles were allowed to vary throughout the cycles of energy minimization. In the last stages of minimization, the penalty distance function was removed and a low-energy structure resembling ribonuclease was obtained (Palmer *et al.*, 1986).

<Glu-Asp-Asn-Ser-Arg-Tyr-Thr-His-Phe-Leu-Thr-Gln-His-Tyr-Asp
-Ala-Lys-Pro-Gln-Gly-Arg-Asp-Asp-Arg-Tyr-Cys-Glu-Ser-Ile-Met
-Arg-Arg-Arg-Gly-Leu-Thr-Ser-Pro-Cys-Lys-Asp-Ile-Asn-Thr-Phe
-Ile-His-Gly-Asn-Lys-Arg-Ser-Ile-Lys-Ala-Ile-Cys-Glu-Asn-Lys
-Asn-Gly-Asn-Pro-His-Arg-Glu-Asn-Leu-Arg-Ile-Ser-Lys-Ser-Ser
-Phe-Gln-Val-Thr-Thr-Cys-Lys-Leu-His-Gly-Gly-Ser-Pro-Trp-Pro
-Pro-Cys-Gln-Tyr-Arg-Ala-Thr-Ala-Gly-Phe-Arg-Asn-Val-Val-Val
-Ala-Cys-Glu-Asn-Gly-Leu-Pro-Val-His-Leu-Asp-Gln-Ser-Ile-Phe
-Arg-Arg-Pro-OH

**Figure 1** Amino acid sequence of human angiogenin (Strydom *et al.*, 1985).

**Table II** Comparison of sequence homologies of human angiogenin and pancreatic ribonuclease

| | *Residues* |
|---|---|
| Identical to human RNase | 43 |
| Identical to other RNase | 25 |
| Conservative replacements | 16 |
| Homology: 84/123 | 68% |

*Ribonucleolytic activity of angiogenin*

Despite its high degree of homology with pancreatic ribonuclease, and despite the use of very sensitive assay methods under a variety of reaction conditions, angiogenin has not been found to be active toward any of the conventional substrates of that enzyme (Shapiro *et al.*, 1986). In particular, it does not produce detectable amounts of acid-soluble products from high molecular weight wheat germ RNA, poly(C), or poly(U), nor does it catalyze the hydrolysis of cytidine- or uridine cyclic 2′3′-phosphates. However, it is active against 28S and 18S ribosomal RNA when tested by measuring their disappearance on agarose gel electrophoresis. This activity differs significantly from that of pancreatic RNase in two respects: (a) it requires $10^4$–$10^5$ as much angiogenin to achieve the same degree of rRNA degradation as with ribonuclease and (b) the fragments generated are relatively large, containing from 100 to 500 nucleotides, compared with those formed by ribonuclease.

The base-cleavage specificity of angiogenin toward RNA has has been determined with purified 5S RNAs from *Saccharomyces cerevisiae* and *Escherichia coli* (Rybak & Vallee, 1988). As with ribonuclease, phosphodiester bond cleavage occurs exclusively at the 3′ side of cytidylic or uridylic acid residues, preferably when the pyrimidine is followed by adenine. However, angiogenin does not cleave after every pyrimidine in the 5S RNAs and the overall pattern of cleavage is not the same as for RNase. There is no evidence for a specific recognition element in terms of the primary sequence of nucleotides in the 5S RNAs. Hence, it seems likely that some aspect of secondary structure may influence endonucleolytic specificity. Analysis of limit digests by low voltage electrophoresis indicates that the regions least susceptible to cleavage are those that derive from RNA segments where stable, base-paired structures are presumed to form.

*Inhibition of cell-free protein synthesis*

The importance of RNA secondary structure for the ribonucleolytic specificity of angiogenin is further evident from its effects on cell-free protein synthesis by the rabbit reticulocyte lysate system (St. Clair *et al.*, 1987). This system was examined to ascertain whether or not angiogenin acts preferentially on one or another class of RNA molecules. Remarkably, the results demonstrated that angiogenin is a potent and, indeed, specific inhibitor of protein synthesis. When incubated with the lysate at a concentration of 40–60 nM, angiogenin completely abolishes protein synthesis. This inhibition is due to the ribonucleolytic activity of angiogenin, since it is accompanied by generation of limited cleavage products from reticulocyte RNA and the effect is inhibited by human placental ribonuclease inhibitor.

The principal target of angiogenin in the reticulocyte lysate is ribosomal RNA. Addition of intact ribosomes to an angiogenin-treated lysate restores the capacity of the lysate to support protein synthesis but addition of mRNA or tRNA does not. However, angiogenin-treated lysosomes are unable to restore this capacity. Moreover, the isolated ribosomes can be resolved into 40S and 60S subunits which can be treated separately with angiogenin. Loss of protein synthesis only occurs on treatment of the small subunits, not the large subunits.

Significantly, neither the 5.8S nor 5S rRNA species are degraded by angiogenin when they are present in the ribosome although, as indicated above, they are susceptible once purified. Moreover, while angiogenin, at 40–60 nM, totally abolishes protein synthesis, this concentration appears to have little effect on 18S and 28S rRNA. This is consistent with kinetic studies which indicate that angiogenin inhibits either the chain elongation or termination step of protein synthesis, not initiation. Therefore, inactivation of only a few ribosomes would be necessary to block protein synthesis completely (Eller *et al.*, 1984). When 10 to 100 times more angiogenin is added to the reticulocyte lysate the 5S and 5.8S rRNAs are still unaffected, but now both the 28S and 18S rRNAs undergo cleavage. The former is broken down into low molecular weight fragments but the latter is transformed into a 230-base product that resists further degradation. Presumably it is this limited nucleolytic process that affects protein synthesis since 18S RNA is a constituent of the angiogenin-sensitive 40S ribosomal subunit.

### *Ribonucleolytic activity and angiogenesis*

The sequence homology to the pancreatic ribonucleases, the ribonucleolytic activity toward isolated RNA, and the potent inhibition of cell-free protein synthesis all raise the question of the relationship of the enzymatic activity of angiogenin to its biological role in angiogenesis. Chemical modification of angiogenin with bromoacetate destroys both the ribonucleolytic and the angiogenic activity of angiogenin virtually completely (Shapiro *et al.*, 1987*b*). Similarly, as discussed below, all of its activities are abolished on treatment with human placental ribonuclease inhibitor, an effect that may have important physiologic implications (Shapiro & Vallee, 1987). It would appear, therefore, that the ribonucleolytic potential of angiogenin is critical to the process of angiogenesis.

Angiogenin is, of course, an extracellular protein. It is secreted into the external medium by tumour cells growing in culture and is present in human plasma at a concentration of $400 \mu g l^{-1}$. Under these circumstances, however, angiogenin is neither cytotoxic nor does it stimulate uncontrolled blood vessel proliferation. Moreover, angiogenin has been added to the growth medium of a wide variety of cell types and in no instance has it been found to be cytotoxic. Whether or not other factors may mediate its internalization under special conditions is not as yet known.

### *Placental ribonuclease inhibitor*

Homology considerations have provided the opportunity to examine the capacity of known inhibitors of pancreatic RNase to inactivate angiogenin. Protein inhibitors of pancreatic and other RNases have been identified in the cytoplasm of a wide variety of mammalian tissues (Blackburn *et al.*, 1977). In particular, the human placental inhibitor, PRI, has been purified and isolated: it very effectively inhibits pancreatic RNase. PRI is also a potent antagonist of both the angiogenic and ribonucleolytic activities of angiogenin (Table III). In fact, it is almost 60 times more effective against angiogenin compared to RNase and the stoichiometry of the interaction is 1:1. Analogous to bovine pancreatic RNase, this inhibition is reversible, and *p*-chloro- mercuribenzoate dissociates the complex readily to yield active angiogenin, chromatographically identical to the native protein. A slight molar excess of PRI suffices to inhibit enzymatic activity completely (Shapiro & Vallee, 1987). Further, carboxymethylation of Lys-41 of pancreatic RNase substantially reduces binding strength, indicating that this residue is essential to the interaction between PRI and RNase A (Lee, F.S., personal communication).

These findings represent a hitherto unsuspected function for PRI: regulation of angiogenesis. This could lead to its therapeutic use in the management of pathological conditions characterized by or dependent on abnormal neovascularization. Inhibition of both the biological and enzymatic activities of angiogenin by human PRI may therefore have important mechanistic, physiological and pharmacologic implications.

In summary, the complete chemical characterization of a unique human organogenic messenger molecule, ie, one that induces organ formation, accomplishes a first major objective in a long-term investigation of organogenesis in general and angiogenesis in particular. The unexpected homology to ribonuclease A allows the use of novel approaches to the investigation of the biological process of angiogenesis and may be relevant to the evolution of organogenic molecules. Importantly, it provides a chemical basis for the prediction of angiogenic function. These provocative findings are thought to have important physiological implications.

**Table III** Inhibition of angiogenin by human placental ribonuclease inhibitor

| *Activity* | *−PRI* | *+PRI* |
|---|---|---|
| RNA hydrolysis[a] | 100 | 0 |
| Angiogenesis, CAM[b] | 57 | 16 |
| Protein synthesis inhibition[c] | 100 | 0 |

[a]RNA (12 μg) was incubated with 0.8 μM angiogenin with or without 0.96 μM PRI at 37°C in 0.033 M HEPES, 0.033 M NaCl, pH 7.5 for 30 min. RNA hydrolysis (%) was determined by agarose gel electrophoresis (Shapiro & Vallee, 1987). [b]Assays were performed on the chick chorioallantoic membrane (CAM) as described (Fett *et al.*, 1985). Results (% positive) are the average of three separate experiments with angiogenin concentrations of 75, 46 and 25 ng, respectively, with and without 2000, 700, and 180 ng PRI, respectively (Shapiro & Vallee, 1987). [c]Protein synthesis was carried out *in vitro* using the rabbit reticulocyte lysate system. Angiogenin, 40 nM, added to the lysate inhibited protein synthesis (100%). Preincubation of angiogenin with an equimolar concentration of PRI abolished the ability of angiogenin to inhibit protein synthesis (St. Clair *et al.*, 1987).

### References

ALDERMAN, E.M., LOBB, R.R. & FETT J.W. (1985). Isolation of tumour-secreted products from human carcinoma cells maintained in a defined serum-free medium. *Proc. Natl Acad. Sci. USA*, **82**, 5771.

BEINTEMA, J.J., WIETZES, P., WEICKMAN, J.L. & GLITZ, D.G. (1984). The amino acid sequence of human pancreatic ribonuclease. *Anal. Biochem.*, **36**, 48.

BEINTEMA, J.J., BROOS, J., MENLENBERG, J. & SCHULLER, C. (1985). The amino acid sequence of snapping turtle (Chelydra serpentina) ribonuclease. *Eur. J. Biochem.*, **153**, 305.

BLACKBURN, P., WILSON, G. & MOORE, S. (1977). Ribonuclease inhibitor from human placenta. Purification and properties. *J. Biol. Chem.*, **252**, 5904.

BLACKBURN, P. & MOORE, S. (1982). Pancreatic ribonuclease. In *The Enzymes*, 3rd ed., Vol. 15, Boyer, P.D. (ed) p. 317. Academic Press: New York.

ELLER, M.S., CULLINAN, R.E. & McGUIRE, P.M. (1984). *Arch. Biochem. Biophys.*, **232**, 526.

FETT, J.W., STRYDOM, D.J., LOBB, R.R. & 4 others (1985). Isolation and characterization of angiogenin, an organogenic protein from human carcinoma cells. *Biochemistry*, **24**, 5480.

FOLKMAN, M.J. & COTRAN, R.S. (1976). Relation of vascular proliferation to tumour growth. *Int. Rev. Exp. Path.*, **16**, 207.

FOLKMAN, J. & KLAGSBRUN, M. (1987). Angiogenic factors. *Science*, **235**, 442.

KURACHI, K., DAVIE, E.W., STYRDOM, D.J., RIORDAN, J.F. & VALLEE, B.L. (1985). Sequence of the cDNA and gene for angiogenin, a human angiogenesis factor. *Biochemistry*, **24**, 5494.

NEEDHAM, J. (1936). New advances in the chemistry and histology of organized growth. *Proc. R. Soc. Med.*, **29**, 1577.

PALMER, K.A., SCHERAGA, H.A., RIORDAN, J.F. & VALLEE, B.L. (1986). A preliminary three-dimensional structure of angiogenin. *Proc. Natl Acad. Sci. USA.*, **83**, 1965.

RYBAK, S.M. & VALLEE, B.L. (1988). Base cleavage specificity of angiogenin with *S. cerevisiae* and *E. coli* 5S RNAs. *Biochemistry*, **27**, 2288.

ST. CLAIR, D.K., RYBAK, S.M., RIORDAN, J.F. & VALLEE, B.L. (1987). Angiogenin abolishes cell-free synthesis by specific ribonucleolytic inactivation of ribosomes. *Proc. Natl Acad. Sci. USA*, **84**, 8330.

SHAPIRO, R. & VALLEE, B.L. (1987). Human placental ribonuclease inhibitor abolishes both angiogenin and ribonucleolytic activities of angiogenin. *Proc. Natl Acad. Sci., USA*, **84**, 2238.

SHAPIRO, R., RIORDAN, J.F. & VALLEE, B.L. (1986). Characteristic ribonucleolytic activity of human angiogenin. *Biochemistry*, **25**, 3527.

SHAPIRO, R., STRYDOM, D.J., OLSON, K.A. & VALLEE, B.L. (1987*a*). Isolation of angiogenin from normal human plasma. *Biochemistry*, **26**, 5141.

SHAPIRO, R., WEREMOWICZ, S., RIORDAN, J.F. & VALLEE, B.L. (1987*b*). Ribonucleolytic activity of angiogenin: essential histidine, lysine and arginine residues. *Proc. Natl Acad. Sci. USA*, **84**, 8783.

SPEMANN, H. & MANGOLD, H. (1924). Uber induktion von embryonalanlagen durch implantation artfremder organistoren. *Arch. Mikrosk. Anat. Entwicklungsmech.*, **100**, 599.

STRYDOM, D.J., FETT, J.W., LOBB, R.R. & 4 others (1985). Amino acid sequence of human tumour derived angiogenin. *Biochemistry*, **24**, 5486.